# Two new species of *Acanthobothrium* Blanchard, 1848 (Onchobothriidae) in *Narcineentemedor* Jordan & Starks, 1895 (Narcinidae) from Acapulco, Guerrero, Mexico

**DOI:** 10.3897/zookeys.852.28964

**Published:** 2019-06-05

**Authors:** Francisco Zaragoza-Tapia, Griselda Pulido-Flores, Juan Violante-González, Scott Monks

**Affiliations:** 1 Universidad Autónoma del Estado de Hidalgo, Centro de Investigaciones Biológicas, Apartado Postal 1-10, C.P. 42001, Pachuca, Hidalgo, México Universidad Autónoma del Estado de Hidalgo Pachuca Mexico; 2 Universidad Autónoma de Guerrero, Unidad Académica de Ecología Marina, Gran Vía Tropical No. 20, Fraccionamiento Las Playas, C.P. 39390, Acapulco, Guerrero, México Universidad Autónoma de Guerrero Acapulco Mexico

**Keywords:** *
Acanthobothrium
*, Elasmobranchii, Helminth, *
Narcine entemedor
*, Onchoproteocephalidea, Torpediniformes

## Abstract

Two species of *Acanthobothrium* (Onchoproteocephalidea: Onchobothriidae) are described from the spiral intestine of *Narcineentemedor* Jordan & Starks, 1895, in Bahía de Acapulco, Acapulco, Guerrero, Mexico. Based on the four criteria used for the identification of species of *Acanthobothrium*, *A.soniae***sp. nov.** is a Category 2 species (less than 15 mm in total length with less than 50 proglottids, less than 80 testes, and with the ovary asymmetrical in shape). *Acanthobothriumvidali***sp. nov.** is a Category 6 species (more than 15 mm in total length with more than 50 proglottids, fewer than 80 testes, and the ovary is asymmetrical). The new species differ from similar species from the Pacific Ocean by total length, the number of proglottids, diameter of the accessory sucker, the length of the cirrus sac, the number of testes per proglottid and the measurements of hooks. With the recognition of *A.soniae***sp. nov.** and *A.vidali***sp. nov.**, 42 species of *Acanthobothrium* have been reported from the Pacific coast of the Americas. This is the first report of species of *Acanthobothrium* from a member of *Narcine* from Mexico and it brings the number of species reported from elasmobranchs from the Pacific Coast of Mexico to 13.

## Introduction

*Acanthobothrium* Blanchard, 1848 is one of the richest genera within Onchoproteocephalidea (Maleki et al. 2013; Caira et al. 2017), but relatively few occurrences have been documented in Mexico. To date, the best-studied locality is the Gulf of California (Sea of Cortez; Mar de Cortés, Golfo de California) with the descriptions of 10 species (Appy and Dailey 1973; Caira and Burge 2001; Caira and Zahner 2001; Ghoshroy and Caira 2001). The only other species known from the Pacific Coast of Mexico was described from the more southern state of Jalisco (Monks et al. 1996) (see Merlo-Serna and García-Prieto 2016). More recently, *A.cartagenensis* Brooks & Mayes, 1980 was reported from Quintana Roo, Mexico (Caribbean) (Monks et al. 2015a) and *A.marquesi* was described from Campeche, Mexico (Gulf of Mexico) by Rodríguez-Ibarra et al. (2018). As part of a collaborative project to extend the knowledge of the helminth fauna of marine fishes in Mexico, rays were collected from the coastal waters off Acapulco, Guerrero, a region with few studies of the parasites of rays. There are six reports of parasites of rays from Acapulco, none for *Acanthobothrium* (see Merlo-Serna and García-Prieto 2016). In this paper, two new species of *Acanthobothrium* are described from *Narcineentemedor* Jordan & Starks, 1895 (Elasmobranchii: Torpediniformes: Narcinidae); one Category 2 species (Ghoshroy and Caira 2001) and one Category 6 species. The new species constitute the first records from the Southern Pacific Coast of Mexico and the first record of species of *Acanthobothrium* reported in *Narcineentemedor* from Mexico.

## Materials and methods

Eleven recently killed specimens of *Narcineentemedor* Jordan & Starks, 1895 (Giant electric ray or Cortez Numbfish) were purchased from local fishermen at Playa Las Hamacas, Bahía de Acapulco, Guerrero (16°51'10.80"N, 99°53'59.02"W) in February, April, May, June 2011 and June 2012. Rays were transported to the laboratory (Universidad Autónoma de Guerrero, Unidad Académica de Ecología Marina) in ice chests containing ice where the spiral intestine was removed and opened by longitudinal incision. Collection, preservation and mounting follow Monks et al. (2015b). Stained specimens were examined using a compound photomicroscope (Leica DM-LB2) equipped with both normal light optics and differential interference contrast (DIC-Nomarski) optics. Figures were drawn with the aid of a drawing tube. Measurements are presented as ranges and number of specimens from which the measurements were taken is given in parentheses. Hook measurements follow Euzet (1959) as modified by Ghoshroy and Caira (2001). Measurements are in micrometers unless otherwise stated. Other hook terminology follows that of Caira (1985). Designation of proglottid apolysis follows Caira et al. (1999) and Franzese and Ivanov (2018). Microtriches on the scolex and peduncle were not examined using SEM, so they are referred to by the general name “microtriches” without qualifying them as proposed by Chervy (2009). The categorical method suggested by Ghoshroy and Caira (2001) and Fyler and Caira 2006 was used to facilitate comparisons among species of *Acanthobothrium* from the Eastern Pacific Ocean and other congeners with similar morphological characters described in the Pacific Ocean. Specimens from several museums were examined, the acronyms are as follows:

**CNHE** (Colección Nacional de Helmintos del Instituto de Biología, Universidad Nacional Autónoma de México, México);

**HWML** (University of Nebraska State Museum, Harold W. Manter Laboratory, Division of Parasitology, Lincoln, Nebraska, USA);

**CHE** (Colección de Helmintos, Centro de Investigaciones Biológicas, Universidad Autónoma del Estado de Hidalgo, Pachuca, México).

Type material was deposited in CNHE (holotype and paratypes), HWML (paratypes), and CHE (paratypes). Application and validity of scientific names, authorities, and common names of fish are in accord with Froese and Pauly (2018) and Last et al. (2016).

## Systematic accounts

### Order Onchoproteocephalidea Caira, Jensen, Waeschenbach, Olson & Littlewood, 2014

#### Family Onchobothriidae Braun, 1900

##### Genus *Acanthobothrium* Blanchard, 1848

###### Type species *Acanthobothriumcoronatum* (Rudolphi, 1819) Blanchard, 1848

####### Acanthobothrium soniae sp. nov.

Taxon classificationAnimaliaOnchoproteocephalideaOnchobothriidae

http://zoobank.org/4E0635D5-3E38-4FEC-8192-4B0AEF53C76C

Figures 1A–E ; 2A–D

######## Type material.

Holotype (CNHE-11136), 3 paratypes (CNHE-11137), 3 paratypes (HWML-139978), and 1 paratype (CHE-P00081).

######## Other material examined.

*Acanthobothriumbullardi* Ghoshroy & Caira, 2001 (CNHE–4046, México) paratype; *A.campbelli* Marques, Brooks & Monks, 1995 (CNHE–3033, Costa Rica; HWML–38546, Costa Rica) voucher and paratype; *A.costarricense* Marques, Brooks & Monks, 1995 (CNHE–3034, Costa Rica) 2 vouchers; *A.dasi* Ghoshroy & Caira, 2001 (CNHE–4044, México; HWML–15549, 15550, 15551, México) 4 paratypes; *A.franus* Marques, Centritto & Stewart, 1997 (CNHE–3140, Costa Rica) paratype; *A.inbiorium* Marques, Centritto & Stewart, 1997 (CNHE–3138, Costa Rica) paratype; *A.puntarenasense* Marques, Brooks & Monks, 1995 (CNHE–4176, Costa Rica) paratype; *A.rajivi* Ghoshroy & Caira, 2001 (CNHE–4039, México) paratype; *A.vargasi* Marques, Brooks & Monks, 1995 (HWML 38545, Costa Rica).

######## Type host.

*Narcineentemedor* Jordan & Starks, 1895 (Elasmobranchii: Torpediniformes: Narcinidae).

######## Type locality.

Bahía de Acapulco (Playa Las Hamacas: 16°51'11"N, 99°53'59"W), Guerrero, México.

######## Site of infection.

Spiral intestine.

######## Quantitative descriptors of parasite populations (Bush et al. 1997).

Prevalence= 9.0% (1 ray of 11 was infected); abundance = 0.73 (8 helminths in 11 rays); mean intensity = 8 (8 helminths in 1 infected ray).

######## Etymology.

The species is named in remembrance of Sonia Virginia Flores León, former player of the Pumas Club Women’s Basketball Team, UNAM, daughter of Virginia León-Règagnon and Martín Ignacio Flores-Carbajal and dear friend of SM and GP-F; she will not be forgotten.

######## Diagnosis.

*Acanthobothriumsoniae* sp. nov. is a Category 2 species. It is small, with a range of 10–13 acraspedote proglottids. The testes are wider than long with a range of 31–47 testes per proglottid. The arms of the ovary are unequal (asymmetrical). Finally, this species also can be distinguished from similar congeners by total length, number of proglottids, diameter of accessory sucker, the length of the cirrus sac, number of testes per proglottid, and size of the hooks.

######## Description.

**Figure 1.**
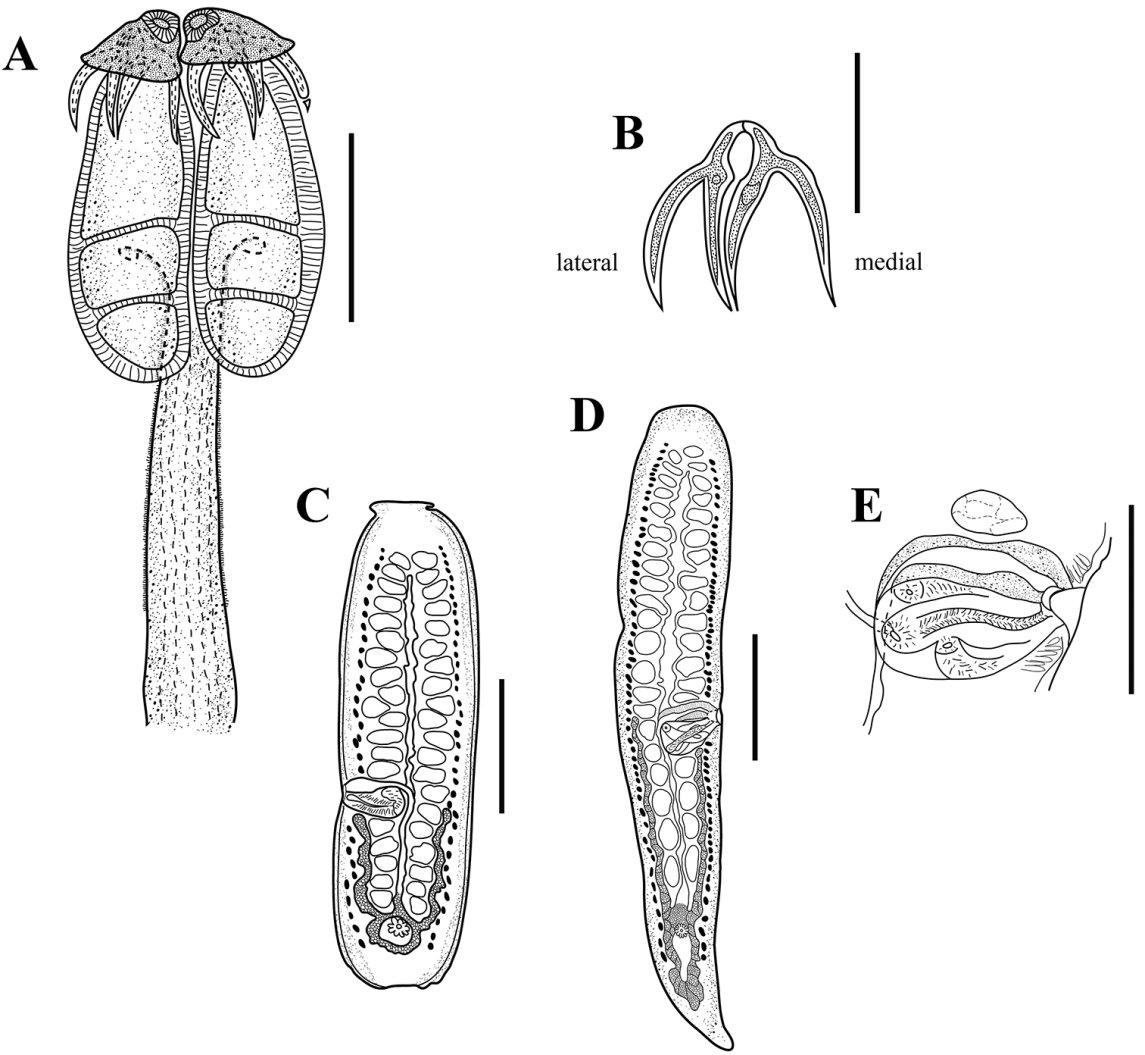
Holotype of *Acanthobothriumsoniae* sp. nov. (CNHE-11136). **A** Scolex **B** hooks **C** mature proglottid **D** terminal proglottid **E** genitalia. Scale bars: 200 µm (**A, D**); 100 µm (**B, E)**; 150 µm (**C**).

**Figure 2.**
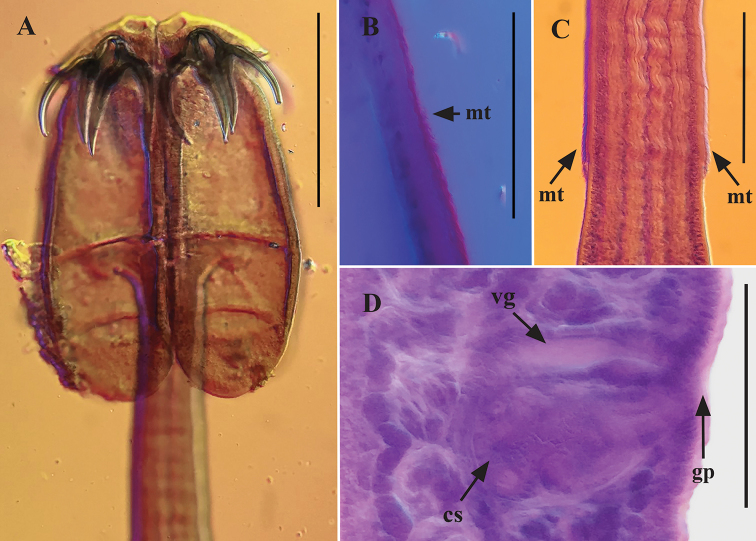
Light microscope photographs of holotype of *Acanthobothriumsoniae* sp. nov. (CNHE-11136). **A** Scolex **B** details of bothridium **C** peduncle cephalic **D** genitalia. Scale bars: 200 µm (**A**); 40 µm (**B**), 100 µm (**C, D**). Abbreviations: mt microtriches; vg vagina; cs cirrus sac; gp genital pore.

[Based on 5 complete worms and 3 partial specimens] Worms 2.9–6.7 mm (n = 5) long, euapolytic; 10–13 (n = 5) proglottids per worm. Scolex 380–420 (n = 6) long by 280–320 (n = 6) wide, with four bothridia. Maximum width of scolex at level of middle loculus (Figs 1A, 2A). Bothridia free posteriorly, tri-locular, 340–380 (n = 6) long by 140–160 (n = 6) wide, with anterior muscular pad (Figs 1A, 2A). Muscular pad 105–130 (n = 6) wide, with apical sucker 45–50 (n = 6) and one pair of bipronged hooks at posterior margin (Figs 1A, 2A). Anterior loculus of bothridia 175–205 (n = 6) long; middle loculus 60–90 (n = 6) long; posterior loculus 80–95 (n = 6) long (Figs 1A, 2A); loculus length ratio (anterior:middle:posterior) 1:0.38:0.46. Velum between medial margins of bothridia in dorsal or ventral pairs not seen (Figs 1A, 2A). Hooks bipronged, hollow, with tubercle on proximal surface of axial prong; internal channels of axial and abaxial prongs continuous, smooth, the base and anterior part of each hook embedded in musculature of scolex, tips of prongs free (Figs 1A, B, 2A). Bases (handles) of medial and lateral hooks articulate to one another (Figs 1B, 2A). Lateral hook measurements (n = 6): A 43–45, B 88–105, C 83–93, D 125–138; Medial hook measurements (n = 6): A’ 38–45, B’ 83–108, C’ 80–98, D’ 125–143. Cephalic peduncle 450–630 (n = 6) long by 70–95 (n = 6) wide, not all the cephalic peduncle is covered with prominent microtriches (Figs 1A, 2C). Scolex is covered with microtriches (Fig. 2B). Proglottids acraspedote. Immature proglottids 55–110 (n = 8) long by 60–115 (n = 8) wide, mature proglottids 225–800 (n = 8) long by 125–215 (n = 8) wide (Fig. 1C), terminal proglottids 585–1,425 (n = 7) long by 160–275 (n = 7) wide (Fig. 1D). Genital pore marginal, irregularly alternating, 56%–68% (n = 7) from anterior end of proglottid; genital atrium present (Fig. 1E). Testes in single layer, arranged in two irregular columns, one on each side of the uterus (Fig. 1C, D). Testes generally wider than long in mature proglottids, 25–63 (n = 8) long by 13–28 (n = 8) wide (Fig. 1C). Total number of testes 31–47 (n = 8), aporal 16–26 (n = 8), poral 15–21 (n = 8), preporal 11–17 (n = 8), and postporal 3–5 (n = 8); all testes located anterior to ovarian isthmus. Cirrus sac pyriform, extending anteriorly (Figs 1C, D, E, 2D), 55–90 (n = 6) long by 63–96 (n = 6) wide in mature proglottids, 85–140 (n = 6) long by 48–90 (n = 6) wide in termial proglottids. Cirrus armed.

Vagina extending laterally from common genital atrium, following anterior margin of cirrus sac, weakly sinuous posteriorly along medial line of proglottid to oötype (Figs 1C, D, E, 2D); vaginal sphincter absent. Seminal receptacle not seen. Ovary inverted A-shaped in frontal view in mature and terminal proglottids (Fig. 1C, D). Arms of ovary unequal (Fig. 1C, D); aporal arm always longer than poral arm. Aporal arm 78–275 (n = 7) long in mature proglottids, 243–625 (n = 7) long in terminal proglottids; poral arm 68–213 (n = 7) long in mature proglottids, 190–550 (n = 7) long in terminal proglottids and Mehlis’ gland posterior to ovarian isthmus. Vitellarium follicular form lateral bands, extending from near anterior margin of proglottid to near posterior margin of proglottid (Fig. 1C, D); follicles 15–23 (n = 6) long by 10–13 (n = 6). Uterus thick-walled, saccate, extending from anterior margin of proglottid to near posterior margin of proglottid. Excretory ducts laterally. Gravid proglottids and eggs not seen.

######## Remarks.

There are 42 Category 2 species (sensu Ghoshroy and Caira 2001) of *Acanthobothrium* that have been described worldwide. Of these, 17 species have been found in the Pacific Ocean, 14 of which are amphi-American (Table 1).

*Acanthobothriumsoniae* sp. nov. is a Category 2 species (sensu Ghoshroy and Caira 2001): Category 2 species have a total length ≤ 15 mm (the length of *A.soniae* sp. nov. is 2.9–6.7 mm), a strobila made up of ≤ 50 proglottids (*A.soniae* sp. nov. has 10–13 proglottids), the number of testes per proglottids ≤ 80 (*A.soniae* sp. nov. has 31–47 testes per proglottid), and the arms of the ovary are asymmetrical.

As presented in Table 1, the new species can be distinguished from similar Category 2 species of *Acanthobothrium* that have been described from the Pacific Coast of the Americas (amphi-American species), and from others parts of Pacific Ocean by the measurements given in the Table 1. The total length of the new species (2.9–6.7 mm) is shorter than that of *A.campbelli* (0.99–1.8 mm). The number of proglottids of the new species (10–13) is less than that of *A.annapinkiensis* Carvajal & Goldstein, 1971 (15–26), *A.cimari* Marques, Brooks & Monks, 1995 (14–33), *A.puntarenasense* (23–27), *A.guanghaiense* Yang, Sun, Zhi, Iwaki, Reyda & Yang, 2016 (13–28), *A.masnihae* Fyler & Caira, 2006 (23–43), *A.popi* Fyler, Caira & Jensen, 2009 (14–20), and *A.tetabuanense* Reyda & Caira, 2006 (25–36), and the number of proglottids of the new species is greater than that of *A.campbelli* (3–6) and *A.vargasi* (5–7). The diameter of the accessory sucker of the new species (45–50) is shorter than that *A.annapinkiensis* (~120) and *A.popi* (50–88), and the diameter of the accessory sucker of the new species is longer that *A.campbelli* (16–38), *A.coquimbensis* Carvajal & Jeges, 1980 (16–44), *A.olseni* Dailey & Mudry, 1968 (24–34), *A.puntarenasense* (14–15), *A.vargasi* (22–41), *A.guanghaiense* (28–32) and *A.popi* (50–88). The length of the cirrus sac of the new species (55–90) is shorter than that of *A.brachyacanthum* Risen, 1955 (123–135), *A.bullardi* (113–175), *A.cimari* (148–180), *A.coquimbensis* (200–400), *A.costarricense* (100–236), *A.dasi* (100–153), *A.olseni* (96–168), *A.puntarenasense* (151–183), *A.unilateralis* Alexander, 1953 (150–160), *A.guanghaiense* (95–132), and *A.popi* (108–152). The number of testes per proglottid of the new species (31–47) is less than that of *A.campbelli* (15–23), *A.rajivi* (9–13), *A.vargasi* (6–12), and *A.tetabuanense* (6–12). Finally the measurements of the hooks of the 18 species can be found in Table 1.

**Table 1.** Comparison of *Acanthobothriumsoniae* sp. nov. vs. Category 2 species of the genus from the Pacific Ocean. Abbreviations: No. Number; A Base (handle) length; B Axial prong length; C Abaxial prong length; D Total hook length. Note: the use of “–” without numerical values are measurement ranges that overlap those of *A.soniae* sp. nov.

Species of *Acanthobothrium*	Total length (mm)	No. of proglottids	Diameter of accesory sucker (μm)	Length of cirrus sac (μm)	No. of testes per proglottid	Measurements of hook (μm)			
						A	B	C	D
**Pacific coast of the Americas**									
*A.soniae* sp. nov.	2.9–6.7	10–13	45–50	55–90	31–47	43–45	88–105	83–93	125–138
*A.annapinkiensis* Carvajal & Goldstein, 1971	–	15–26	~ 120	–	–	60–80	180–250	160–240	240–310
*A.brachyacanthum* Riser, 1955	–	–	–	123–135	–	–	66	57–60	90
*A.bullardi* Ghoshroy & Caira, 2001	–	–	–	113–175	–	–	–	43–78	–
*A.campbelli* Marques, Brooks & Monks, 1995	0.99–1.8	3–6	16–38	–	15–23	16–41	–	–	95–120
*A.cimari* Marques, Brooks & Monks, 1995	–	14–33	–	148–180	–	–	–	57–82	–
*A.coquimbensis* Carvajal & Jeges, 1980	–	–	16–44	200–400	–	–	–	96–136	–
*A.costarricense* Marques, Brooks & Monks, 1995	–	–	–	110–236	–	–	–	54–66	–
*A.dasi* Ghoshroy & Caira, 2001	–	–	–	100–153	–	–	–	50–75	68–125
*A.olseni* Dailey & Mudry, 1968	–	–	24–34	96–168	–	–	–	–	91–115
*A.puntarenasense* Marques, Brooks & Monks, 1995	–	23–27	14–15	151–183	–	–	72–82	75–81	107–114
*A.rajivi* Ghoshroy & Caira, 2001	–	–	–	–	9–13	28–35	63–73	58–68	88–98
*A.unilateralis* Alexander, 1953	–	–	–	150–160	–	58–64	118	118	173–182
*A.vargasi* Marques, Brooks & Monks, 1995	–	5–7	22–41	–	22–29	–	–	–	–
**Other parts of the Pacific Ocean**									
*A.guanghaiense* Yang, Sun, Zhi, Iwaki, Reyda & Yang, 2016	–	13–28	28–32	95–132	–	–	–	94–124	–
*A.masnihae* Fyler & Caira, 2006	–	23–43	–	–	6–12	–	69–88	–	99–123
*A.popi* Fyler, Caira & Jensen, 2009	–	14–20	50–88	108–152	–	–	–	–	–
*A.tetabuanense* Reyda & Caira, 2006	–	25–36	–	–	6–12	–	–	–	–

####### Acanthobothrium vidali sp. nov.

Taxon classificationAnimaliaOnchoproteocephalideaOnchobothriidae

http://zoobank.org/9D9106EF-7772-4E61-A44F-B2D151782329

Figures 3A–D ; 4A–D

######## Type material.

Holotype (CNHE-11134), 7 paratypes (CNHE-11135), 3 paratypes (HWML-139979, 139980, 139981), and 7 paratypes (CHE-P00082).

######## Other material examined.

*Acanthobothriumfranus* Marques, Centritto & Stewart, 1997 (CNHE–3140, Costa Rica) paratype; *A.inbiorium* Marques, Centritto & Stewart, 1997 (CNHE–3138, Costa Rica) paratype; *A.obuncus* Marques, Brooks & Barriga, 1997 (CNHE–3032A, 3167B, Ecuador) holotype; *A.soberoni* Ghoshroy & Caira, 2001 (CNHE–4042, México).

######## Type host.

*Narcineentemedor* Jordan & Starks, 1895 (Elasmobranchii: Torpediniformes: Narcinidae).

######## Type locality.

Bahía de Acapulco (Playa Las Hamacas: 16°51'11"N, 99°53'59"W), Guerrero, México.

######## Site of infection.

Spiral intestine.

######## Quantitative descriptors of parasite populations (Bush et al. 1997).

Prevalence = 36.36% (4 of 11 rays were infected); abundance = 1.91 (21 helminths in 11 rays); mean intensity = 5.25 (21 helminths in 4 infected rays).

######## Etymology.

The species is named in honor of Dr Victor Vidal Martínez (Departamento de Recursos del Mar, CINVESTAV-IPN, Merida, Yucatan, Mexico), for his contribution to our knowledge of helminths of fishes from Mexico.

######## Diagnosis.

*Acanthobothriumvidali* sp. nov. is a Category 6 species. This species is large, with a range of 164–214 craspedote proglottids, with a range of 50–76 testes per proglottid, and the arms of ovary unequal in length (asymmetrical). This new species also can be distinguished from similar congeners by total length, number of proglottids, diameter of accessory sucker, the length of the cirrus sac, number of testes per proglottid, and size of the hooks.

######## Description.

**Figure 3.**
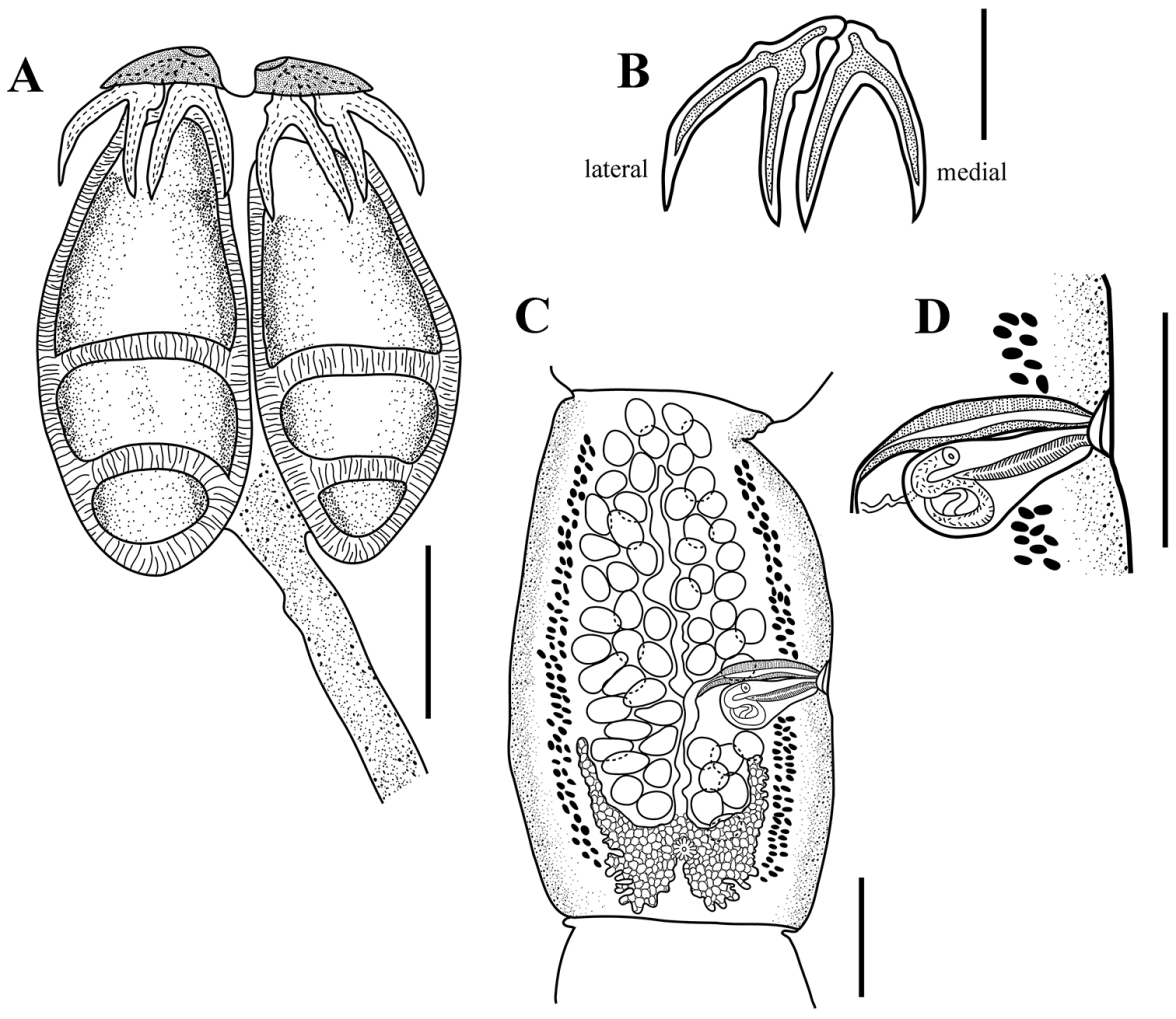
Holotype of *Acanthobothriumvidali* sp. nov. (CNHE-11134). **A** Scolex **B** hooks **C** mature proglottid **D** genitalia. Scale bars: 400 µm (**A**); 200 µm (**B–D**).

**Figure 4.**
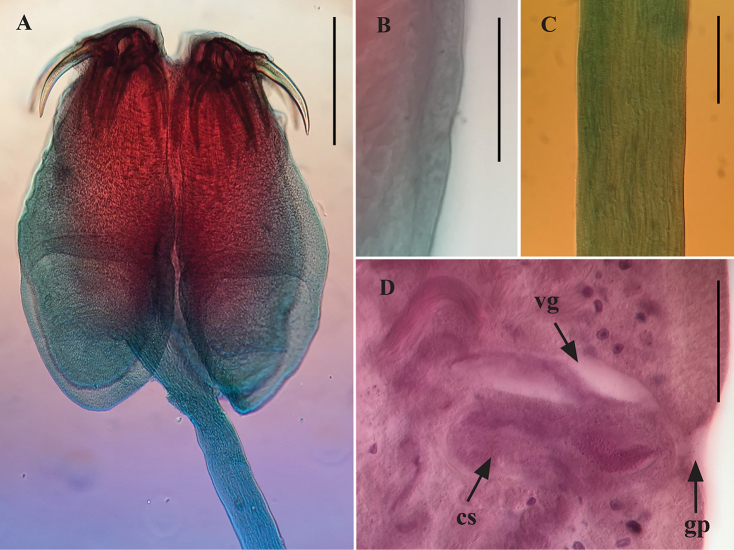
Light microscope photographs of holotype of *Acanthobothriumvidali* sp. nov. (CNHE-11134). **A** Scolex **B** details of bothridium **C** peduncle cephalic **D** genitalia. Scale bars: 400 µm (**A**); 40 µm (**B**);100 µm (**C, D**). Abbreviations: vg vagina; cs cirrus sac; gp genital pore.

[Based on 5 complete worms and 16 partial specimens] Worms 26.5–70.9 mm (n = 5) long, greatest width at level of mature proglottids, euapolytic; 164–214 (n = 5) proglottids per worm. Scolex 880–1,400 (n = 20) long by 680–1,170 (n = 20) wide, with four bothridia; maximum width of scolex at level of posterior margin of anterior loculus (Figs 3A, 4A). Bothridia free posteriorly, tri-locular, 770–1,230 (n = 20) long by 320–570 (n = 20) wide, with anterior muscular pad (Figs 3A, 4A). Muscular pad 250–325 (n = 18) wide, with apical sucker 75–150 (n = 19) and one pair of bipronged hooks at posterior margin (Figs 3A, 4A). Anterior loculus of bothridia 400–650 (n = 20) long; middle loculus 170–310 (n = 20) long; posterior loculus 150–340 (n = 20) long (Figs 3A, 4A); loculus length ratio (anterior:middle:posterior) 1:0.48:0.50. Velum between medial margins of bothridia in dorsal or ventral pairs not seen (Figs 3A, 4A). Hooks bipronged, hollow, with tubercle on proximal surface of axial prong; internal channels of axial and abaxial prongs continuous, smooth, base and anterior part of each hook embedded in musculature of scolex, tips of prongs free (Figs 3A, B, 4A). Bases (handles) of medial and lateral hooks articulate with one another (Figs 3B, 4A). Lateral hook measurements (n = 15): A 140–170, B 200–285, C 140–305, D 360–465; Medial hook measurements (n = 15): A’ 100–165, B’ 225–300, C’ 200–270, D’ 300–425. Cephalic peduncle 2.38–9.13 mm (n = 15) long by 0.15–0.23 mm (n = 15) wide, microtriches not seen on the scolex or cephalic peduncle (Figs 3A, 4A, B, C). Proglottids craspedote. Immature proglottids 50–230 (n = 17) long by 240–520 (n = 17) wide, mature proglottids 260–700 (n = 10) long by 300–790 (n = 10) wide (Fig. 3C), terminal proglottids 1,120 (n = 1) long by 480 (n = 1) wide. Genital pore marginal, irregularly alternating, 49%–63% (n = 9) of proglottid length from anterior end in mature proglottids; genital atrium present (Figs 3C, D, 4D). Testes arranged in two to three irregular columns on each side of the uterus, in frontal view testes wider than long in mature proglottids, 50–125 (n = 6) long by 40–50 (n = 6) wide (Fig. 3C). In terminal proglottids, anteriormost testes wider than long and posteriormost testes longer than wide. Total number of testes 50–76 (n = 10), aporal 26–40 (n = 10), poral 23–36 (n = 10), preporal 17–26 (n = 10), postporal 5–11 (n = 10). All testes located anterior to ovarian isthmus. Cirrus sac pyriform, 125–175 (n = 6) long by 30–75 (n = 6) wide in mature proglottids (Figs 3C, D, 4D). Cirrus armed. Vagina anterior to cirrus sac (Figs 3C, D, 4D),walls relatively thick, covered with gland cells. Vagina extending laterally from common genital atrium, following anterior margin of cirrus sac, weakly sinuous posteriorly along medial line of proglottid to oötype (Fig. 3C); vaginal sphincter absent. Seminal receptacle not seen. Ovary in mature proglottids H-shaped in frontal view (Fig. 3C); posterior lobes wider than anterior lobes. Ovarian isthmus approximately 2/3 of the distance from anterior end of ovary. Arms of ovary unequal in length, aporal arm always longer than poral arm (Fig. 3C). Aporal arm 150–260 (n = 8) long, reaching to posterior margin of cirrus sac, poral arm 125–225 (n = 8) long in mature proglottids, not reaching posterior margin of cirrus sac. Mehlis’ gland posterior to ovarian isthmus. Vitiellarium follicular, forming lateral bands, extending from near anterior margin of proglottid to near posterior margin of proglottid (Fig. 3C); follicles 15–20 (n = 8) long by 10–15 (n = 8) wide. Uterus thin-walled, saccate, extending from anterior margin of proglottid to near posterior margin of proglottid. Excretory ducts lateral. Gravid proglottids and eggs not seen.

######## Remarks.

There are 14 Category 6 species of *Acanthobothrium* that have been described worldwide. Of these, seven species have been found in the Pacific Ocean, four of which are amphi-American (Table 2). *Acanthobothriumvidali* sp. nov. also is a Category 6 species, bringing the total number to 15. Category 6 species have a total length >15 mm (the length of *A.vidali* sp. nov. is 26.5–70.9 mm), a strobila made up of > 50 proglottids (*A.vidali* sp. nov. has 164–214 proglottids), number of testes per proglottids ≤ 80 (*A.vidali* sp. nov. has 50–76 testes per proglottid), and the arms of the ovary are asymmetrical.

As presented in Table 2, the new species can be distinguished from similar Category 6 species of *Acanthobothrium* that have been described from the Pacific Coast of the Americas (amphi-American species), and from others parts of Pacific Ocean by the measurementes given in Table 2. The total length of the new species (26.5–70.9 mm) is longer than that of *A.aetiobatidis* (Shipley, 1900) Southwell, 1925. The number of proglottids of the new species (164–214) is greater than that of *A.gonzalesmugaburoi* Severino & Sarmiento, 1979. The diameter of accessory sucker of the new species (75–150) is larger than that of *A.obuncus* (33–48) and *A.soberoni* (40–65). The length of the cirrus sac of the new species (125–175) is shorter than that of *A.obuncus* (258–322), *A.aetiobatidis* (200–250), and *A.rodmani* Fyler, Caira & Jensen, 2009 (190–234). The number of testes per proglottid of the new species (50–76) is greater than that of *A.aetiobatidis* (23–28), *A.arlenae* Campbell & Beverage, 2002 (17–26), and *A.rodmani* (17–26). Finally, the measurements of the hooks of the eight species can be found in Table 2.

The new species is the fourth species of *Acanthobothrium* reported from *N.entemedor*, preceded by *A.franus* and *A.inbiorium* (Category 5 species), and *A.soniae* sp. nov. (Category 2 species) described above. All species have been reported from the Pacific Coast of the Americas. *Acanthobothriumvidali* sp. nov. can be distinguished from these other species by number of proglottids (164–214) is greater than that of *A.franus* (68–141) and *A.soniae* sp. nov. (10–13). The total length of the lateral hook of *A.vidali* sp. nov. (360–465) is longer than that of *A.inbiorium* (95–120 µm). The length of the axial prong of the lateral hook of *A.vidali* sp. nov. (200–285) is longer than that of *A.inbiorium* (65–75 µm) (Table 3).

**Table 2.** Comparison of *Acanthobothriumvidali* sp. nov. vs. Category 6 species of the genus from the Pacific Ocean. Abbreviations: No. Number; A Base (handle) length; B Axial prong length; C Abaxial prong length; D Total hook length. Note: the use of “–” without numerical values are measurement ranges that overlap those of *A.vidali* sp. nov.

Species of *Acanthobothrium*	Total length (mm)	No. of proglottids	Diameter of accesory sucker (μm)	Length of cirrus sac (μm)	No. of testes per proglottid	Measurements of hook (μm)			
						A	B	C	D
**Pacific coast of the Americas**									
*A.vidali* sp. nov.	26.5–70.9	164–214	75–150	125–175	50–76	140–170	200–285	140–305	360–465
*A.gonzalesmugaburoi* Severino & Sarmiento, 1979	–	38–63	–	–	–	50–87	67–123	57–120	146–219
*A.maculatum* Riser, 1955	–	–	–	–	–	72–78	72–78	75–78	135–141
*A.obuncus* Marques, Brooks & Barriga, 1997	–	–	33–48	258–322	–	66	60–64	63–64	126–130
*A.soberoni* Ghoshroy & Caira, 2001	–	–	40–65	–	–	45–88	43–90	65–100	86–158
**Other parts of the Pacific Ocean**									
*A.aetiobatidis* (Shipley, 1900), Southwell, 1925	15–20	–	–	200–250	23–28	–	120–130	120–130	250–280
*A.arlenae* Campbell & Beveridge, 2002	–	–	–	–	17–26	–	160–179	–	289–344
*A.rodmani* Fyler, Caira & Jensen, 2009	–	–	–	190–234	17–26	–	–	–	335–357

**Table 3.** Comparison of species of *Acanthobothrium* that have been reported from *Narcineentemedor*. Abbreviations: No. Number; A Base (handle) length; B Axial prong length; C Abaxial prong length; D Total hook length. Note: Information taken from the original descriptions and this study.

Species of *Acanthobothrium*	Total length (mm)	No. of proglottids	Diameter of accesory sucker (μm)	Length of cirrus sac (μm)	No. of testes per proglottid	Measurements of hook (μm)			
						A	B	C	D
**Reported from *Narcineentemedor***									
*A.franus* Marques, Centritto & Stewart, 1997*	16.0–40.0	68–141	60–159	102–281	24–59	118–175	245–319	223–322	354–465
*A.inbiorium* Marques, Centritto & Stewart, 1997	28.0–82.0	156–223	20–75	122–285	44–73	35–50	65–75	50–60	95–120
*A.soniae* sp. nov.	2.9–6.7	10–13	45–50	55–90	31– 47	43–45	88–105	83–93	125–138
*A.vidali* sp. nov.	26.5–70.9	164–214	75–150	125–175	50– 76	140–170	200–285	140–305	360–465

## Discussion

To date, 190 valid species of *Acanthobothrium* have been reported from different regions of the world (Caira et al. 2017; Rodríguez-Ibarra et al. 2018; Franzese and Ivanov 2018). Forty species of *Acanthobothrium* have been described from the Pacific coast of the Americas (eleven species from USA, eleven from México, eight from Costa Rica, four from Ecuador, four from Peru, and three from Chile). With these descriptions of *A.soniae* sp. nov. and *A.vidali* sp. nov., 13 species of *Acanthobothrium* have been reported from the Pacific Coast of Mexico. A list of amphi-American species of *Acanthobothrium* from the Pacific coast, their hosts, and localities is given in Table 4.

**Table 4.** Species of *Acanthobothrium* reported from the Pacific Ocean of the Americas (amphi-American species). ‡= Category designation obtained from Ghoshroy and Caira (2001). Category designations not included in Ghoshroy and Caira (2001) were calculated for this study using the original descriptions. Sources were as given by that author or the original descriptions used for this study.

Family / Host species	*Acanthobothrium* species	Type locality	Source	Category designation
** Heterodontidae **				
*Heterodontusfrancisci* (Girard, 1855)	*A.bajaensis* Appy & Dailey, 1973	San Quintin Bay, Baja California, Mexico	Appy and Dailey (1973)	**4**‡
	*A.puertecitense* Caira & Zahner, 2001	Puertecitos, Gulf of California, Mexico	Caira and Zahner (2001)	**4**
*H.mexicanus* Taylor & Castro-Aguirre, 1972	*A.santarosaliense* Caira & Zahner, 2001	Santa Rosalia, Gulf of California, Mexico	Caira and Zahner (2001)	**3**
** Rhinobatidae **				
*Pseudobatosproductus* (Ayres, 1854)	*A.olseni* Dailey & Mudry, 1968	Newport Beach, California, USA	Dailey and Mudry (1968)	**2**‡
	*A.rhinobati* Alexander, 1953	Santa Monica Harbor, California, USA	Alexander (1953)	**9(5)**‡
	*A.robustum* Alexander, 1953	Long Beach Harbor, California, USA	Alexander (1953)	**4**‡
** Platyrhinidae **				
*Platyrhinoidistriseriata* (Jordan & Gilbert, 1880)	*A.goldsteini* Appy & Dailey, 1973	Seal Beach, California, USA	Appy and Dailey (1973)	**5(9)**‡
** Narcinidae **				
*Diplobatisommata* (Jordan & Gilbert, 1890)	*A.dollyae* Caira & Burge, 2001	Bahía de Los Angeles, Gulf of California, Mexico	Caira and Burge (2001)	**1**
	*A.maryanskii* Caira & Burge, 2001	Loreto, Gulfo of California, Mexico	Caira and Burge (2001)	**5**
	*A.royi* Caira & Burge, 2001	Punta Arena, Gulf of California, Mexico	Caira and Burge (2001)	**1**
*Narcineentemedor* Jordan & Starks, 1895	*A.franus* Marques, Centritto & Stewart, 1997	Cuajiniquil Beach, Gulf of Santa Helena, Guanacaste, Costa Rica	Marques et al. (1997b)	**5(8)**‡
	*A.inbiorium* Marques, Centritto & Stewart, 1997	Cuajiniquil Beach, Gulf of Santa Helena, Guanacaste, Costa Rica	Marques et al. (1997b)	**5**‡
	*A.soniae* sp. n.	Playa las Hamacas, Bahía de Acapulco, Guerrero, Mexico	This study	**2**‡
	*A.vidali* sp. n.	Playa las Hamacas, Bahía de Acapulco, Guerrero, Mexico	This study	**6**‡
** Torpedinidae **				
*Tetronarcecalifornica* (Ayres, 1855)	*A.hispidum* Riser, 1955	Monterey Bay, California, USA	Riser (1955)	**5**‡
** Rajidae **				
*Rajastellulata* (Gilbert, 1915)	*A.brachyacanthum* Riser, 1955	Monterey Bay, California, USA	Riser (1955)	**2**‡
*Zearajachilensis* (Guichenot, 1848)	*A.annapinkiensis* Carvajal & Goldstein, 1971	Anna Pink Hay, Chile	Carvajal-G. and Goldstein (1971)	**2**‡
** Arhynchobatidae **				
*Psammobatisscobina* (Philippi, 1857)	*A.psammobati* Carvajal & Goldstein, 1969	South Pacific Ocean, between Papudo and Talcahuano, Chile	Carvajal-G. and Goldstein (1971)	**5**‡
*Sympterygiabrevicaudata* (Cope, 1877)	*A.lusarmientoi* Severino & Verano, 1980	Callao, Lima, Peru	Severino and Verano (1980)	**7**
** Gymnuridae **				
*Gymnuraafuerae* (Hildebrand, 1946)	*A.atahualpai* Marques, Brooks & Barringa, 1997	Puerto Bolivar, Provincia de El Oro, Ecuador	Marques et al. (1997a)	**1**‡
** Dasyatidae **				
*Hypanusdipterurus* (Jordan & Gilbert, 1880)	*A.bullardi* Ghoshroy & Caira, 2001	Bahía de Los Angeles, Gulf of California, Mexico	Ghoshroy and Caira (2001)	**2**‡
	*A.dasi* Ghoshroy & Caira, 2001	Puertecitos, Gulf of California, Mexico	Ghoshroy and Caira (2001)	**2**‡
	*A.rajivi* Ghoshroy & Caira, 2001	Puertecitos, Gulf of California, Mexico	Ghoshroy and Caira (2001)	**2**‡
	*A.soberoni* Ghoshroy & Caira, 2001	Puertecitos, Gulf of California, Mexico	Ghoshroy and Caira (2001)	**6**‡
*H.longus* (Garman, 1880)	*A.cimari* Marques, Brooks & Monks, 1995	Punta Morales, Puntarenas Province, Costa Rica	Marques et al. (1995)	**2**‡
	*A.cleofanus* Monks, Brooks & Lonce de Leon, 1996	Chamela Bay, Jalisco, Mexico	Monks et al. (1996)	**3**‡
	*A.costarricense* Marques, Brooks & Monks, 1995	Punta Morales, Puntarenas Province, Costa Rica	Marques et al. (1995)	**2**‡
	*A.obuncus* Marques, Brooks & Barriga, 1997	Puerto Hualtaco, Provincia de El Oro, Ecuador	Marques et al. (1997a)	**6**‡
	*A.puntarenasense* Marques, Brooks & Monks, 1995	Punta Morales, Puntarenas Province, Costa Rica	Marques et al. (1995)	**2**‡
	*A.vargasi* Marques, Brooks & Monks, 1995	Punta Morales, Puntarenas Province, Costa Rica	Marques et al. (1995)	**2**‡
** Potamotrygonidae **				
*Potamotrygonmotoro* (Müller & Henle, 1841)	*A.peruviense* Reyda, 2008	Madre de Dios River at Boca Manu, Madre de Dios Department, Peru	Reyda (2008)	**1(8)**
** Urotrygonidae **				
*Urobatishalleri* (Cooper, 1863)	*A.parviuncinatum* Young, 1954	San Diego Bays, California, USA	Young (1954)	**8**‡
*U.tumbesensis* (Chirichigno F. & McEachran, 1979)	*A.minusculus* Marques, Brooks & Barriga, 1997	Puerto Hualtaco, Provincia de El Oro, Ecuador	Marques et al. (1997a)	**1**‡
*Urotrygonchilensis* (Günther, 1872)	*A.campbelli* Marques, Brooks & Monks, 1995	Costa de Pajaros, Puntarenas, Costa Rica	Marques et al. (1995)	**2**‡
** Myliobatidae **				
*Myliobatiscalifornicus* Grill, 1865	*A.holorhini* Alexander, 1953	Long Beach Harbor, California, USA	Alexander (1953)	**3**‡
	*A.maculatum* Riser, 1955	Monterey Bay, California, USA	Rêgo et al. 1968	**6(3)**‡
	*A.microcephalum* Alexander, 1953	Long Beach Harbor, California, USA	Alexander (1953)	**4**‡
	*A.unilateralis* Alexander, 1953	Long Beach Harbor, California, USA	Alexander (1953)	**7(2)**‡
*M.chilensis* Philippi, 1892	*A.coquimbensis* Carvajal & Jeges, 1980	Antofagasta, Chile	Carvajal-G. and Jeges-G. (1980)	**2**‡
*M.peruvianus* Garman, 1913	*A.gonzalesmugaburoi* Severino & Sarmiento, 1979	Callao, Lima, Peru	Severino and Sarmiento (1979)	**7(6)**
** Aetobatidae **				
*Aetobatusnarinari* (Euphrasen, 1790)	*A.monksi* Marques, Brooks & Barriga, 1997	Puerto Jelí, Provincia de El Oro, Ecuador	Marques et al. (1997a)	**1**‡
	*A.nicoyaense* Brooks & McCorquodale, 1995	Punta Morales, Golfo de Nicoya, Costa Rica	Brooks and McCorquodale (1995)	**1**‡
**Scombridae (Perciformes)**				
*Sardachiliensis* (Cuvier, 1832)	*A.chilensis* Rego, Vincednte & Herrera, 1968	Paita, Piúra, Peru	Rêgo et al. (1968)	**3**‡

Host specificity of most species of *Acanthobothrium* appears to be rather strict (Ivanov, 2005; Vardo-Zalik and Campbell 2011; Franzese and Ivanov 2018). According to the reports of species of the genus (type localities, additional localities, type host, and additional host), 82% of the species of *Acanthobothrium* show strict host specificity. In contrast, 33 of the 190 valid species of *Acanthobothrium* have been reported in more than one species of host (see the reports of Rudolphi 1819; Yoshida 1917; MacCallum 1921; Léon-Borcéa 1935; Baer 1948; Euzet 1952; Yamaguti 1952; Young 1954; Riser 1955; Rees and Williams 1965; Goldstein 1967; Campbell 1969; Williams 1969; Carvajal-G. and Jeges-G. 1980; Rodriguez and Tantaleán-Vidaurre 1980; Brooks et al. 1981; Mayes and Brooks 1981; Escalante-A. 1986; Tantaleán-Vidaurre 1991; Marques et al. 1997a; Campbell and Beveridge 2002; Friggens and Brown 2005; Lacerda et al. 2008; and, Iannacone et al. 2011).

Prior to de Carvalho and Last (2016), the genus *Narcine* Henle, 1834 was composed of 20 species. To date, those taxa have been divided into two genera; 15 species of *Narcine* (tail length about equal to disc length or width) and 5 species of *Narcinops* de Carvalho & Last, 2016 (tail much longer tan disc length or width), this latter distributed only in Australia (Last et al. 2016). No helminths have been reported from the former members of *Narcine* that are now assigned to *Narcinops*. Five valid species of *Acanthobothrium* have been reported worldwide from three species of *Narcine* (Table 5) (Subhapradha, 1955; Goldstein et al. 1969; Brooks and Mayes 1978; Marques et al. 1997b), but no species of *Acanthobothrium* in *Narcine* have been reported from Mexico (Merlo-Serna and García-Prieto 2016). In Mexico, only two species of helminth have been reported previously from *Narcine*: *Anaporrhutumeuzeti* Curran, Blend & Overstreet, 2003 and *Nagmiarodmani* Curran, Blend & Overstreet, 2009 (Curran et al. 2003; Curran et al. 2009).

**Table 5.** Species of *Acanthobothrium* reported in species of *Narcine*. † Data from Ghoshroy and Caira (2001); ‡ Data from Fyler and Caira (2006).

* Narcine *	Species of	Category	Habitat of host	Type locality	Source
*Narcine* sp. (Reported as *N.braunii*, synonym of *N.brasiliensis*)	*A.indicum* Subhapradha, 1955	‡ 5	Northern Indian Ocean	Madras Coast, India	Subhapradha (1955)
*N.bancroftii* (Griffith & Smith, 1834) [reported as *N.brasiliensis* (Olfers, 1831)]	*A.lintoni* Goldstein, Henson & Schlicht, 1968	† 1(8,9,5)	North Carolina to northeastern Brazil	Gulf of Mexico, Texas, USA	Goldstein et al. (1969)
* N. brasiliensis *	*A.electricolum* Brooks & Mayes, 1978	† 9	Brazil to northern Argentina	Caribbean Sea, near Cartagena, Colombia	Brooks and Mayes (1978)
*N.entemedor* Jordan & Starks, 1895	*A.franus* Marques, Centritto & Stewart, 1997	† 5(8)	Baja California to northen Peru	Cuajiniquil Beach, Gulf of Santa Helena, Guanacaste, Costa Rica	Marques et al. (1997b)
	*A.inbiorium* Marques, Centritto & Stewart, 1997	† 5	Baja California to northen Peru	Cuajiniquil Beach, Gulf of Santa Helena, Guanacaste, Costa Rica	Marques et al. (1997b)
	*A.soniae* sp. nov.	2	Baja California to northen Peru	Playa las Hamacas, Bahía de Acapulco, Guerrero, Mexico	This study
	*A.vidali* sp. nov.	6	Baja California to northen Peru	Playa las Hamacas, Bahía de Acapulco, Guerrero, Mexico	This study

The categorical method suggested by Ghoshroy and Caira (2001) and Fyler and Caira, (2006) was used to facilitate comparisons among the 190 valid species of *Acanthobothrium*. Ghoshroy and Caira (2001) proposed the categories to facilitate comparisons among taxa from the same geographic region. Because of the large number of species worldwide, it is necessary to focus only on those species from the same region that possessing the same combination of characters as the new species; thus, delimiting the comparison between similar species that could be confused with a new species and not comparing each new species to all of the 190 valid species currently described. We agree that comparisons with each species of this expanding group is unnecessary and, as the number of species increases, an exercise in futility. In agreement with previous authors, this categorical method is useful but does not reflect groupings from a rigorous phylogenetic hypothesis (i.e., is phenetic) (Campbell and Beveridge 2002; Ivanov 2005; Reyda and Caira 2006; Twohig et al. 2008; Fyler and Caira 2010; Yang et al. 2016).

Although not all species of the genus have been examined, Franzese and Ivanov (2018) suggest that the pattern of microthiches is quite uniform among species of *Acanthobothrium*; (i.e., all species have filitriches covering most surfaces of the worms, interspersed with gladiate spinitriches on proximal bothridial surface, scolex proper and the cephalic peduncle). Because of insufficient material, it was not possible to make a study of this species using the SEM, so we cannot provide detailed information on the microtriches.

## Supplementary Material

XML Treatment for Acanthobothrium soniae

XML Treatment for Acanthobothrium vidali
